# Age, Sex, BMI, Meal Timing, and Glycemic Response to Meal Glycemic Load

**DOI:** 10.1001/jamanetworkopen.2025.33193

**Published:** 2025-09-23

**Authors:** Mar Calvo-Malvar, Óscar Lado-Baleato, Ana Cao Ríos, Cristina Porca Fernández, Alfonso Benítez-Calvo, Carmen Fernandez-Merino, Juan Sánchez-Castro, Robert Wagner, Marcos Matabuena, Francisco Gude

**Affiliations:** 1 Department of Laboratory Medicine, University Clinical Hospital of Santiago de Compostela, Santiago de Compostela, Spain; 2 Research Methodology Group, Health Research Institute of Santiago de Compostela (IDIS), Santiago de Compostela, Spain; 3 ISCIII Instituto de Salud Carlos III Support Platforms for Clinical Research, Health Research Institute of Santiago de Compostela (IDIS), Santiago de Compostela, Spain; 4 Unit of Biostatistics, Department of Statistics, Mathematical Analysis, and Optimization, University of Santiago de Compostela, Santiago de Compostela, Spain; 5 Department of Endocrinology and Nutrition, University Hospital Complex of Ferrol, Ferrol, Spain; 6 Epigenomics in Endocrinology and Nutrition Group, Santiago Health Research Institute (IDIS), Santiago de Compostela, Spain; 7 Department of Radiology, Hospital Lucus Augusti, Lugo, Spain; 8 A Estrada Primary Care Center, A Estrada, Pontevedra, Spain; 9 Department of Psychiatry, Radiology, Public Health, Nursing, and Medicine, University of Santiago, Santiago de Compostela, Spain; 10 Department of Endocrinology and Diabetology, Medical Faculty and University Hospital Düsseldorf, Heinrich Heine University Düsseldorf, Düsseldorf, Germany; 11 Institute for Clinical Diabetology, German Diabetes Center, Leibniz Center for Diabetes Research at Heinrich Heine University Düsseldorf, Düsseldorf, Germany; 12 German Center for Diabetes Research (DZD), Neuherberg, Germany; 13 Department of Biostatistics, Harvard University, Boston, Massachusetts; 14 Primary Care Concepción Arenal, Santiago de Compostela, Spain

## Abstract

**Importance:**

Postprandial glycemic responses contribute to comorbidities and mortality risk, but the association between food and postprandial glucose responses in general population settings remains uncertain.

**Objective:**

To investigate the association of dietary glycemic load (GL), meal timing, age, sex, body mass index (BMI), and glycated hemoglobin (HbA_1c_) concentration with postprandial glycemic response to mixed meals.

**Design, Setting, and Participants:**

This cross-sectional study was conducted from August 21, 2012, to March 26, 2015, at a primary health care center in A Estrada, northwestern Spain. A population-based sample of adults aged 18 to 85 years without diabetes who were randomly selected from National Health System records agreed to participate. Data analysis was performed between April 20, 2023, and March 26, 2024.

**Exposure:**

The main exposure was dietary GL. Additional exposures included age, sex, BMI, meal timing, and HbA_1c_ concentration.

**Main Outcome and Measures:**

Postprandial glucose response over 3 hours after breakfast, lunch, and dinner was assessed using continuous glucose monitoring (CGM) for 7 days, with dietary assessments. Multilevel regression models evaluated the association between GL and glucose dynamics, accounting for age, sex, BMI, meal timing, and HbA_1c_ concentration.

**Results:**

Of the 622 participants fitted with the CGM device, 514 (median age, 46 years [IQR, 36-58 years]; 64% females) met eligibility criteria and provided analyzable data. More than 1.3 million glucose measurements were analyzed across 2451 days. Dietary GL was associated with higher postprandial blood glucose levels, with maximum rises of up to 1.3 (95% CI, 0.8-1.8) mg/dL per 10 units of GL. Glucose responses were greater and more prolonged after lunches and dinners than after breakfasts, with peak values observed at 70 minutes after lunches and dinners and 50 minutes after breakfasts. Each 10-year increase in age was associated with an increase in postprandial glucose levels of 1.9 (95% CI, 0.6-3.3) mg/dL to 3.5 (95% CI, 2.2-4.8) mg/dL, while BMI was associated with glucose response after breakfast, with increases of up to 0.7 (95% CI, 0.4-1.1) mg/dL per BMI unit increase. Men had lower glucose levels than women during the late postprandial period after lunch and dinner, with differences of up to 4.6 (95% CI, 1.6-7.6) mg/dL. HbA_1c_ concentrations and meal timing were also associated with postprandial glucose levels (eg, glucose levels increased up to 12.0 [95% CI, 6.5-17.5] mg/dL per 1% increase in HbA_1c_).

**Conclusions and Relevance:**

In this cross-sectional study of adults without diabetes, higher-GL meals were associated with sustained postprandial glucose elevations, especially after lunch and dinner. Age, sex, BMI, meal timing, and HbA_1c_ concentration were also associated with glucose responses. These findings support the validity of dietary GL as an explanatory factor for glycemic response to mixed meals under typical everyday conditions when meal timing, age, and BMI are considered.

## Key Points

**Question:**

Are the glycemic load of mixed meals, age, sex, body mass index (BMI), and meal timing associated with postprandial glucose responses in individuals without diabetes?

**Findings:**

In this cross-sectional study of 514 adults without diabetes, meals with higher glycemic load were associated with sustained blood glucose elevation, particularly after lunch and dinner. Age notably modified curves of glucose levels after meals, whereas greater BMI was associated with higher and male sex with lower postprandial glucose levels.

**Meaning:**

These findings suggest that glycemic load is associated with postprandial glucose response in the general population of adults without diabetes, accounting for meal timing, age, sex, and BMI.

## Teaser

This cross-sectional study investigates whether dietary glycemic load, meal timing, and characteristics of age, sex, body mass index, and glycated hemoglobin concentration are associated with postprandial glycemic responses to daily meals.

## Introduction

Postprandial glycemia is a critical factor in overall health, yet no accurate method exists for estimating the glycemic response to food in population-based scenarios. High interindividual variability in response to identical meals highlights the limitations of a one-size-fits-all approach.^1^

The glycemic load (GL), which combines the glycemic index (GI) and available carbohydrate content, has been considered a reliable predictor of glycemic response under standardized conditions.^2^ However, its predictive capacity in free-living conditions remains uncertain.^3^

Beyond their role in predicting postprandial glycemia, low-GI and low-GL diets have been associated with a reduced risk of major chronic diseases and related metabolic risk factors.^3^ Yet, the integration of GI and GL into dietary guidelines remains inconsistent worldwide—some countries regulate GI labeling while others do not,^3^ and the latest World Health Organization guidelines prioritize fiber and whole grains as key indicators of carbohydrate quality.^4^

Although recent studies have reignited the debate over the relevance of GI and GL in chronic disease prevention,^5,6^ evidence of their effects on glycemic response in everyday conditions remains limited. Continuous glucose monitoring (CGM) offers a unique opportunity to capture glycemic response beyond controlled settings, considering the influence of meal timing, food combinations, and individual characteristics.

We hypothesized that investigating the association of dietary and individual factors with postprandial glycemia in typical daily settings using CGM would provide novel insights into the association between GL and glycemic response, addressing existing research limitations. This study addressed 2 key questions: (1) whether dietary GL is associated with postprandial glycemic response in a general population setting and (2) what role meal timing, age, sex, and body mass index (BMI, calculated as weight in kilograms divided by height in meters squared) have in the glycemic response to mixed meals in adults without diabetes using CGM.

## Methods

### Study Design

This research was a cross-sectional substudy of the A Estrada Glycation and Inflammation Study, an epidemiologic investigation designed to assess the association between dysglycemia, inflammation markers, and the risk of diabetes and cardiovascular disease in a general population sample. This substudy was conducted from August 21, 2012, to March 26, 2015, at the local primary health care center in the rural town of A Estrada in northwestern Spain. Procedures are detailed in previous publications.^7^ The current study was approved by the Galician Clinical Research Ethics Committee and followed the principles of the Helsinki Declaration,^8^ with written informed consent obtained from all participants. The research adhered to the Strengthening the Reporting of Observational Studies in Epidemiology (STROBE) reporting guideline. The current data analysis was performed between April 20, 2023, and March 26, 2024.

### Participants

Recruitment for this study involved a random representative sample of individuals aged 18 to 85 years from National Health System records for a rural population of approximately 20 000 inhabitants. Exclusion criteria included pregnancy, alcoholism, major cardiovascular disease, dementia or inability to communicate, a life expectancy of less than 1 year due to a terminal disease, inability to fulfil the study protocol, frequent dining out, allergy to adhesives, or any medical condition likely to affect CGM device performance. Inclusion criteria for participants who underwent a 7-day period of CGM required at least 1 complete day of CGM data, complete food intake records during CGM periods, and no diabetes diagnosis at baseline, following ADA criteria^9^ (eMethods in Supplement 1).

### Procedures

Participants were evaluated at baseline, before CGM device placement. Data collected included sociodemographic characteristics, medical history, tobacco use and alcohol consumption, medications, anthropometrics, and physical activity, and blood samples were collected for laboratory analysis (eFigure 1 in Supplement 1). Instructions for completing a 7-day food intake record coinciding with the CGM period were provided.

At the start of monitoring, a research nurse inserted a subcutaneous sensor (Enlite [Medtronic]) in the participant’s abdomen and provided instructions on device use (iPro2 [Medtronic]). On day 7, the sensor was removed, and CGM data, excluding day 1, were downloaded. Days with more than 2 hours of acquisition failure were excluded from the analysis (eMethods in Supplement 1).

### Outcomes

#### Glycemic Response

Glycemic response was assessed using the iPro system, a professional CGM device that provides glucose profiles retrospectively. The subcutaneous sensor measured interstitial glucose every 5 minutes. Participants used a conventional glucose meter for calibration, performing at least 3 capillary blood glucose measurements per day. Days with fewer than 3 calibrations were excluded to ensure data reliability (eMethods in Supplement 1).

#### Dietary Intake

A 7-day food diary that coincided with the CGM period was used to collect dietary intake data (eMethods in Supplement 1). A nutritionist (C.P.F.) reviewed all records with participants to ensure completeness and accuracy. Dietary intake, including GI and GL, was analyzed using DIAL software, version 3.3.5.0 (Alce Ingeniería),^10^ which uses the Food Composition Tables of the Department of Nutrition at the Complutense University of Madrid.^11^ Energy and nutrient intake are expressed as daily medians.

#### Glycemic Indices

For each main meal (breakfast, lunch, and dinner), the GI and GL were calculated as follows (eMethods in Supplement 1):

GI intake per main meal = ∑(available carbohydrates per food × GI of each food)/total available carbohydrate intake

GL intake per main meal = (total available carbohydrate intake × GI per main meal intake)/100

### Statistical Analysis

Multilevel functional regression models were used to assess changes in glucose concentrations over time, capturing how glycemic response curves evolved and were modified by covariates.^12^ We evaluated interstitial glucose concentration (IGC) during a 3-hour period after each meal (breakfast, lunch, and dinner) for each individual and day. As this was an exploratory study, no specific sample size was predetermined.

To evaluate the explanatory capacity of the multilevel functional model, functional *R*^2^ metrics were applied to quantify the proportion of variance explained across the postprandial period.^13^ Joint *R*^2^ incorporates both fixed and random effects to assess the overall model fit, while marginal *R*^2^ focuses solely on fixed effects, highlighting the influence of potential explanatory factors (eg, GL, age, sex, BMI, glycated hemoglobin [HbA_1c_] level, meal type, and meal timing) independent of individual variability. Model diagnostics were checked using leave-one-out mean absolute error, Pearson residuals, and confidence band coverage of estimated coefficients through parametric bootstrapping (eMethods in Supplement 1). In addition, based on the proposed model, we integrated meal GL with age and BMI to estimate the percentage of time that the blood glucose level exceeded 130 mg/dL (to convert to millimoles per liter, multiply by 0.0555)—as a functional cutoff point^14,15^—during the 3-hour postprandial window after breakfast, lunch, and dinner.

Statistical significance was established for ranges of variables where the estimated 95% global confidence bands did not include 0. All statistical analyses were performed using R, version 4.1.0 (R Project for Statistical Computing)^16^ with the fda and ggplot2 packages.

## Results

### Baseline Characteristics of Study Participants

From a random representative sample of 3500 individuals, 622 participants were fitted with the CGM device for 7 days. Of those, 65 were excluded for diabetes, 4 for nonadherence to daily calibration requirements, 37 for sensor disconnection, and 2 for incomplete or unreliable food records (eFigure 2 in Supplement 1). The final sample included 514 participants (186 men [36%], 328 women [64%]) with a median age of 46 years (range, 18-84 years; IQR, 36-58 years) and median BMI of 27.3 (IQR, 23.9-30.9) (Table). More than 1.3 million glucose measurements were analyzed over 2451 days.

**Table.** Characteristics and Diet of Participants

Characteristic	Participants (N = 514)^a^
**Individuals**	
Age, median (IQR), y	46 (36-58)
Sex	
Men	186 (36)
Women	328 (64)
BMI, median (IQR)	27.3 (23.9-30.9)
FPG, median (IQR), mg/dL	87 (80-94)
HbA_1c_, median (IQR), %	5.3 (5.2-5.5)
HbA_1c_ between 5.7% and 6.4%	121 (24)
Days of device-use data	
5	444 (86)
4	38 (7)
3	17 (3)
2	13 (3)
1	2 (<1)
**Diet**	
Energy intake per meal, median [IQR], kcal (% of total daily intake)	
Breakfast	276.1 [186.4-413.9] (16.6)
Lunch	812.7 [590.5-1072.4] (48.8)
Dinner	575.4 [392.2-843.2] (34.6)
Carbohydrate intake per meal, median (IQR), g	
Breakfast	39.8 (25.3-58.8)
Proportion of total breakfast energy intake, %	57.7
Lunch	65.1 (42.1-96.2)
Proportion of total lunch energy intake, %	32.0
Dinner	51.3 (31.5-78.2)
Proportion of total dinner energy intake, %	35.7
Lipid intake per meal, median (IQR), g	
Breakfast	7.4 (3.2-13.3)
Proportion of total breakfast energy intake, %	24.1
Lunch	34.5 (21.2-53.7)
Proportion of total lunch energy intake, %	38.2
Dinner	24.8 (12.8-39.6)
Proportion of total dinner energy intake, %	38.8
Protein intake, No. (%), g	
Breakfast	10.1 (6.1-14.5)
Proportion of total breakfast energy intake, %	14.6
Lunch	36.7 (25.7-49.8)
Proportion of total lunch energy intake, %	18.1
Dinner	24.0 (15.0-36.5)
Proportion of total dinner energy intake, %	16.7
Physical activity	
Inactive	184 (36)
Minimally active	195 (38)
HEPA active^b^	135 (26)

Abbreviations: BMI, body mass index (calculated as weight in kilograms divided by height in meters squared); FPG, fasting plasma glucose; HbA_1c_, glycated hemoglobin; HEPA, healthy eating and physical activity.

SI conversion factors: To convert FPG to millimoles per liter, multiply by 0.0555; HbA_1c_ to proportion of total hemoglobin, multiply by 0.01.

^a^ Data are presented as number (percentage) of participants unless otherwise indicated.

^b^ Indicates high physical activity that improves health.

### Association of GL, Participant Characteristics, and Meal Type and Timing With Postprandial Glycemic Responses

**Figure 1.**
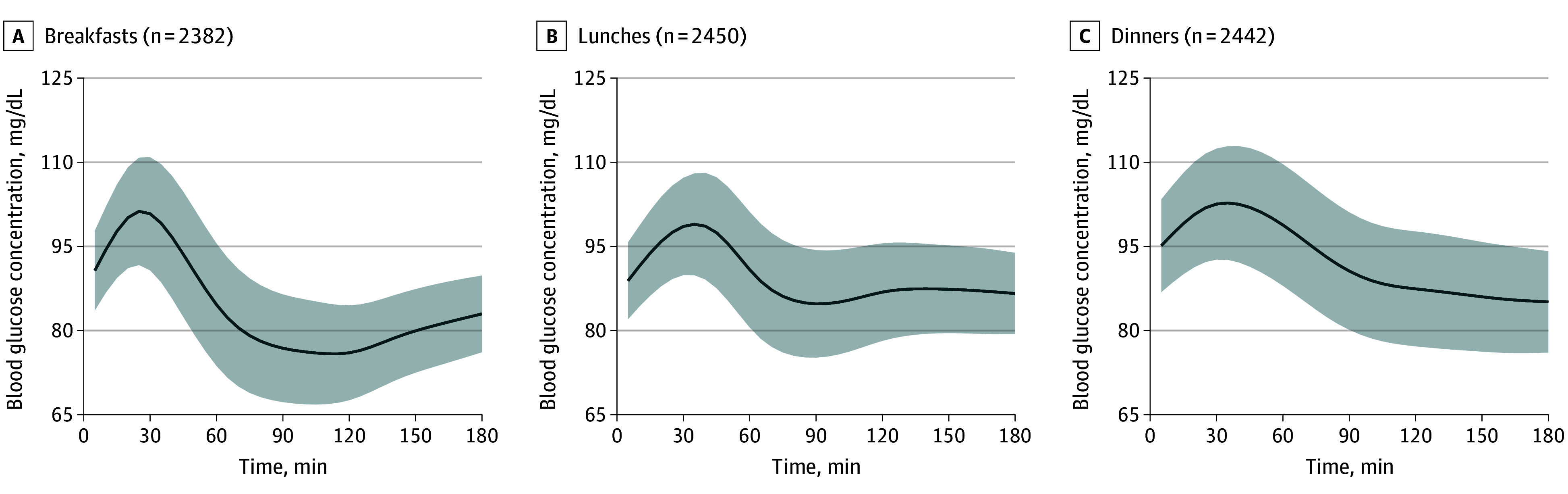
Model Intercepts for Mean Postprandial Interstitial Glucose Levels After Breakfast, Lunch, and Dinner in 514 Participants The solid line represents the mean baseline glucose curve (β_0_) coefficients, adjusted for glycemic load, age, sex, body mass index, glycated hemoglobin level, and meal timing and corresponding to the glucose concentration at a given time. Shading represents the 95% CI. To convert glucose concentration to millimoles per liter, multiply by 0.0555.

**Figure 2.**
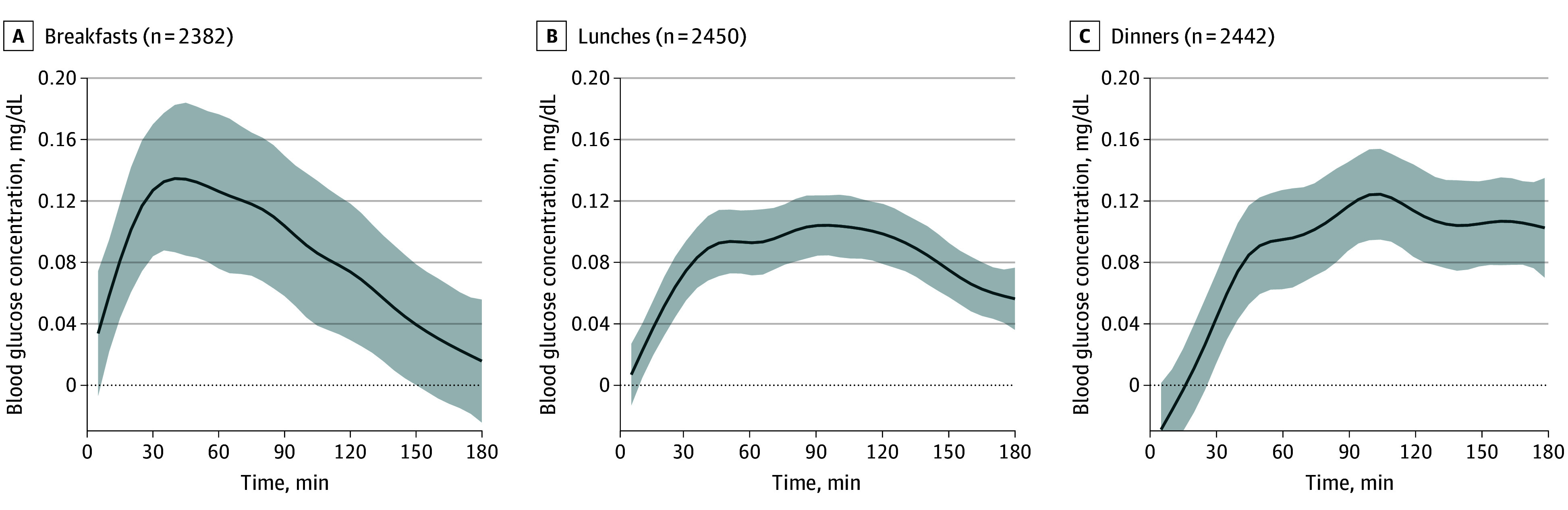
Association of Glycemic Load (GL) With Interstitial Glucose Concentrations by Meal Type The solid line represents the mean glycemic load (β_1_) coefficients during the 3-hour postintake period, indicating the increase in interstitial glucose concentration for each unit increase in GL from the time of intake. To calculate the glucose concentration at a specific time, this value should be multiplied by the corresponding GL from the intake and added to the baseline glucose curve (β_0_) coefficient (which represents the basal glucose concentration) to determine the interstitial glucose concentration at that time. Shading represents the 95% CI. To convert glucose concentration to millimoles per liter, multiply by 0.0555.

**Figure 3.**
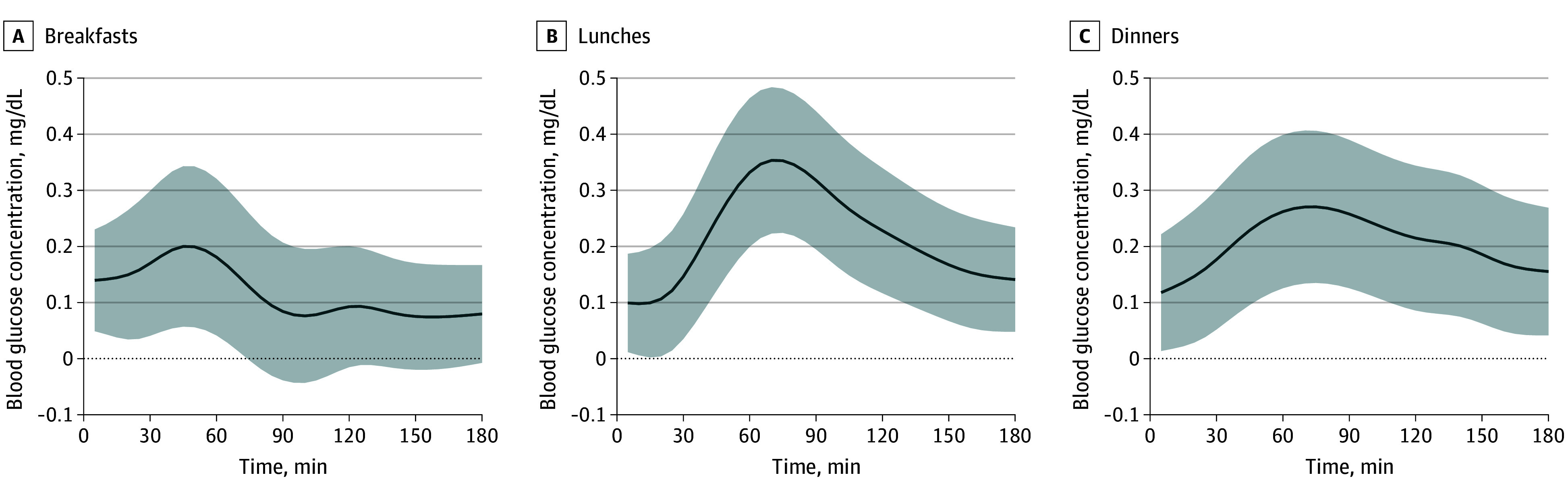
Association of Age With Interstitial Glucose Concentrations by Meal Type The solid line represents the mean age per 1-year increase (β_3_) coefficient values over time after each meal, reflecting the association of age (per 1-year increase) with glucose response. Shading represents the 95% CI. To convert glucose concentration to millimoles per liter, multiply by 0.0555.

**Figure 4.**
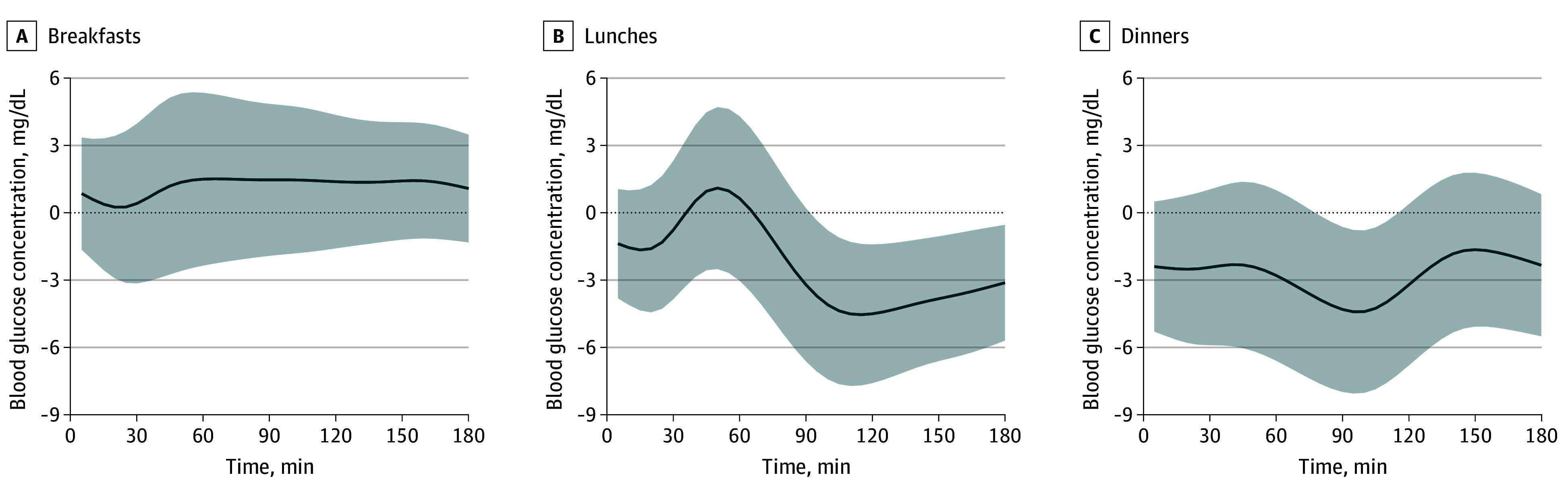
Association of Sex With Interstitial Glucose Concentrations by Meal Type The solid line represents the mean sex (β_4_) coefficient values over time after each meal, reflecting the association between male (compared with female) sex and glucose response. Shading represents the 95% CI. To convert glucose concentration to millimoles per liter, multiply by 0.0555.

A total of 7274 mixed meals (2382 breakfasts, 2450 lunches, and 2442 dinners) were analyzed (eTable 1 in Supplement 1). Figure 1 shows similar postmeal glucose curves, peaking at 20 to 45 minutes after breakfasts, lunches, and dinners, with a faster observed return to baseline after breakfast (50 minutes) than after lunch or dinner (70 minutes). Figure 2, Figure 3, and Figure 4 and eFigures 3 and 4 in Supplement 1 present the associations between GL, age, sex, BMI, HbA_1c_ concentration, meal timing, and CGM measurements by meal type.

#### GL

Participants’ median GL level was 104 units (IQR, 76-142 units) (eTable 1 in Supplement 1). As shown in Figure 2, GL was associated with significantly increased blood glucose levels across all meals. Glucose levels rose up to 1.3 mg/dL (95% CI, 0.8-1.8 mg/dL) per 10-unit GL increase, peaking at approximately 45 minutes after breakfast, 90 minutes after lunch, and 105 minutes after dinner (Figure 2). While breakfast levels returned to baseline, lunch and dinner levels remained elevated for at least 3 hours postmeal. No association was found between glycemic response to GL and absolute intake of protein, fat, or fiber.

#### Age and Sex

Figure 3 illustrates that increasing age was associated with a significant increase in postprandial glucose levels by 1.9 mg/dL (95% CI, 0.6-3.3 mg/dL) to 3.5 mg/dL (95% CI, 2.2-4.8 mg/dL) per decade, with the greatest increase after lunch and the least after breakfast. The increased blood glucose levels associated with age remained consistent throughout the postprandial period for lunches and dinners, while for breakfasts, glucose levels returned to baseline earlier. Men had lower glucose concentrations than women, with differences up to 4.6 mg/dL (95% CI, 1.6-7.6 mg/dL) at lunch and dinner (Figure 4).

#### BMI and HbA_1c_

As depicted in eFigure 3 in Supplement 1, BMI was associated with glucose levels after breakfast, with a maximum increase of 0.7 mg/dL (95% CI, 0.4-1.1 mg/dL) per BMI unit increase. BMI was associated with a smaller increase in glucose levels after lunch and dinner. As expected, HbA_1c_ percentage (to convert to proportion of total hemoglobin, multiply by 0.10) was associated with postprandial glucose concentration, which increased up to 12.0 mg/dL (95% CI, 6.5-17.5 mg/dL) per 1% increase in HbA_1c_ (eFigure 3 in Supplement 1).

#### Meal Type and Timing

The intervals between waking and eating and between dinner and bedtime were associated with postprandial glucose levels. Earlier wake-up time was associated with higher glucose levels after breakfast but lower levels after lunch, while later bedtime was associated with lower postdinner glucose levels (eFigure 4 in Supplement 2).

### Association of Meal GL With Postprandial Glycemic Responses at Different GL, Age, and BMI Values

eFigure 5 in Supplement 1 shows the association of GL and age with postprandial glucose curves. eFigure 6 in Supplement 1 presents the association of GL and BMI with postprandial glucose curves. Higher GL values were associated with greater glucose level elevations after all meals and with more prolonged postprandial glucose levels after lunch and dinner, where levels remained elevated for the entire 3-hour period without returning to baseline. Age appeared to further accentuate this outcome; each decade increase in age was associated with steeper glucose rises after all meals and, for lunch and dinner, a prolonged plateau before a gradual decline (eFigure 5 in Supplement 1). In individuals aged 75 years or older, particularly after lunch and dinner, higher GL values were associated with postprandial glucose levels exceeding 130 mg/dL (eFigure 5 in Supplement 1). BMI exhibited a similar, though less pronounced, pattern (eFigure 6 in Supplement 1).

### Time Above 130 mg/dL

As shown in eFigure 7 in Supplement 1, there was a positive association between GL and time with glucose level above 130 mg/dL postmeal, with individuals older than 60 years experiencing prolonged postprandial hyperglycemia, particularly after dinner. Participants younger than 40 years remained below a glucose level of 130 mg/dL regardless of GL. BMI modified this association, with elevated glucose (>130 mg/dL) observed only after high-GL breakfasts in individuals with higher BMI.

### Model Performance

The explanatory power of the multilevel functional model was assessed using functional *R*^2^ metrics, providing a time-dependent measure of model fit. Breakfasts exhibited the highest joint *R*^2^ (eFigure 8 in Supplement 1), indicating the best overall fit, whereas lunches and dinners showed slightly lower values but a more sustained glucose response, possibly reflecting meal-specific postprandial dynamics.

To further explore model performance, eFigure 9 in Supplement 1 presents the marginal *R*^2^ for different model specifications across meals. The null model, including only random effects, had the lowest explanatory power, while the full model achieved the highest marginal *R*^2^. Meal-specific patterns revealed better performance for breakfasts and lunches, while dinners showed the lowest performance, suggesting a greater influence of unmeasured factors on evening postprandial glucose variability.

eTable 2 in Supplement 1 summarizes key model fit metrics across meals, reinforcing the observed differences in explanatory power. Breakfasts consistently yielded the highest joint *R*^2^, while lunches and dinners exhibited progressively lower values.

Model diagnostics are shown in eTable 2 in Supplement 1. Median mean absolute errors were 5.5 mg/dL, 6.2 mg/dL, and 6.1 mg/dL for breakfasts, lunches, and dinners, respectively. No heteroscedasticity was found in the Pearson residuals (eFigure 10 in Supplement 1). The marginal coverage of the confidence band for each covariate was close to the nominal 95% level (eTable 3 in Supplement 1). In addition, to facilitate interpretation, eTable 4 in Supplement 1 provides a list of study participants’ recorded meals, categorized by their GL values.

## Discussion

In a cohort of 514 adults without diabetes, we analyzed 7274 postprandial glycemic response records from mixed meals under typical daily conditions. We found a statistically significant association between meal GL and postprandial glycemic response, with higher GLs leading to sustained glucose level elevation, particularly after lunch and dinner. Age was consistently associated with increased glucose levels following these meals, while BMI had a significant association only after breakfast. Men exhibited lower glucose levels than women during the late postprandial period after lunch and dinner. Meal timing also was associated with glycemic response, with waking time associated with responses at breakfast and lunch and bedtime associated with postdinner glucose levels. HbA_1c_ level was positively associated with postprandial glucose levels.

Although an increase in postprandial glucose level of up to 1.3 mg/dL per 10 units of GL may seem modest, the IQR of median GL spanned 76 to 142 units. This translates into an average increase of about 10 mg/dL in postprandial glucose level, which in individuals older than 75 years was associated with glucose elevations above 130 mg/dL. This pattern may be clinically relevant, as spending over 10% of time above 130 mg/dL was associated with an increased risk of type 2 diabetes in a recent CGM study of individuals without diabetes.^14^

In our study, postprandial glycemic responses were higher at dinner than at breakfast. We also found that individuals who woke up early exhibited lower glycemic responses at breakfast but higher responses at lunch. In contrast, those with a longer interval between dinner and bedtime showed lower postprandial glucose peaks. These findings align with evidence that circadian rhythms influence glucose metabolism across the day.^17^ Early risers often exhibit greater morning insulin sensitivity, which may explain their lower glycemic response at breakfast.^17^ However, as the day progresses, insulin sensitivity tends to decline, potentially leading to higher postprandial glucose levels at lunch and dinner in early risers.^17,18^

Differences in glycemic response to GL between meals may reflect interactions between carbohydrates and other nutrients in mixed meals. Although carbohydrates are the main drivers of glycemic response,^19^ fats,^20^ proteins,^21^ and fiber^22^ also modulate it, possibly contributing to variability. No association was found with absolute intake of protein, fat, or fiber.

Age was significantly associated with glucose levels across all meals, modifying the associations with GL and glucose curve shapes after lunch and dinner, where glucose levels plateaued. Although aging is associated with β-cell dysfunction and diabetes risk,^23^ few CGM studies have explored its impact in individuals without diabetes.^13,24,25^ Understanding these age-related changes may be crucial for detecting early signs of glycemic deterioration. In this study’s middle-aged cohort, women showed higher postprandial glucose levels than men, possibly due to menopause-related declines in insulin sensitivity.^26^

Greater BMI was associated with increased postprandial glucose levels, though less so than age, with significant associations only after breakfast and in the last 90 minutes postdinner. BMI also modified GL’s association with glycemic response and was associated with altered glucose curve shapes after lunch and dinner, leading to a plateau. Despite BMI being a diabetes risk factor, few CGM studies have examined BMI’s effect on glucose profiles in individuals without diabetes, often using varying metrics.^1,7,14,27,28^ We focused on BMI for consistency with those previous studies, acknowledging that central obesity may also influence glycemic responses.

From a methods standpoint, functional data analysis enabled us to assess the association between individual characteristics (eg, diet, age, sex, and BMI) and CGM-derived glucose trajectories over time, capturing variability across individuals and days while addressing CGM data complexity.^1,2^ This approach accommodated incomplete CGM or meal data, eliminating the need for imputation. Additionally, multilevel models require fewer data and improve statistical power by leveraging multiple observations per individual. Functional *R*^2^ metrics assessed explanatory power over time. While previous studies have examined postprandial glycemic response often focusing on discrete time points,^9^ our approach modeled continuous glucose responses over the full 3-hour postprandial period, providing a more comprehensive representation of the association of diet and other factors with glycemic response.

The shape of the postprandial glucose curve is as relevant as peak levels. CGM-derived metrics such as time above range, linked to diabetes risk independently of HbA_1c_,^2,9^ highlight this. Though not our focus, the finding of sustained increases in glucose levels with higher GL, age, and BMI and in females reinforce the utility of CGM-related metrics in identifying individuals at risk of diabetes, regardless of HbA_1c_ level.

To facilitate functional data visualization, we developed a CGM-based risk table (eFigure 7 in Supplement 1) showing the association between GL, age, and BMI and the percentage of time glucose levels exceeded 130 mg/dL postprandially for each main meal. The 130 mg/dL threshold was chosen as an optimal functional cutoff to identify individuals at increased diabetes risk.^1,3,15^ This table illustrates how age, BMI, and meal type modulated glycemic response to GL. Ultimately, our goal was to model GL’s association with glycemic response while accounting for specific covariates. Further validation is needed to establish its clinical utility.

### Strengths and Limitations

Strengths of this study include the evaluation of a large, randomly selected cohort of adults without diabetes, using ad libitum intake and CGM data over 6 days alongside comprehensive dietary assessment. We intentionally selected a community with moderate socioeconomic and educational levels to enhance generalizability, aligning with Organisation for Economic Co-operation and Development indicators at the time of study design.^29^

Statistically, our use of multilevel functional data analysis enhances understanding of how glucose levels evolve over time as a mathematical function and explores the statistical associations between covariates (eg, GL, age, BMI, and sex) and functional CGM postprandial responses. Model diagnostics indicated that the model assumptions were satisfied and that the model provided a good fit to the data.

Several limitations should be considered when interpreting our findings. First, the cross-sectional design limits conclusions on long-term disease prognosis. However, to our knowledge, no longitudinal study of this scale has used CGM data in a population without diabetes, aside from those previously mentioned.^14,30^ Second, participants willing and able to wear a CGM device and complete food records for 1 week may differ from the general population, potentially limiting generalizability. Third, although 7-day dietary records were reviewed by nutritionists, self-reported dietary intake is subject to recall bias and measurement error, which may attenuate associations. Correction methods such as dietary biomarkers or repeated recalls were not available. Fourth, although physical activity was collected via questionnaire, it was not included in the main models due to limited granularity and its secondary relevance in this study. Nevertheless, as a well-established modifier of glucose metabolism,^31^ omission of physical activity may introduce unmeasured confounding. Fifth, other factors, such as dietary composition, prior glycemia, glucose-modulating medications, and smoking, may influence glycemic response and warrant further study.

## Conclusions

In this cross-sectional study of adults without diabetes, we found that glycemic response to meals was associated with the GL of the intake, supporting the validity of this index as an explanatory factor for glycemic response in mixed meals under typical everyday conditions. Meal timing, age, sex, and BMI were associated with the glycemic responses and with both the magnitude and shape of the postprandial glucose curves. These findings support the validity of dietary GL as an explanatory factor for glycemic response to mixed meals under typical everyday conditions when meal timing, age, and BMI are considered.

## Notes

Supplement 1.
eMethods.
**eFigure 1.** Study Procedures **eFigure 2.** Flowchart of the Recruitment Process and Study Phases **eTable 1.** Participants’ Daily Intake and GL Distribution **eFigure 3.** Effect of BMI and HbA_1c_ on Postprandial Glucose Trajectories **eFigure 4.** Effect of Meal Timing on Postprandial Glucose Trajectories **eFigure 5.** Effect of GL on Postprandial Glucose Across Different Age Values **eFigure 6.** Effect of GL on Postprandial Glucose Across Different BMI Values **eFigure 7.** Percentage of Postprandial Time Spent Above 130 mg/dL Depending on GL, BMI, and Age for Each Meal **eFigure 8.** Estimated Joint *R*^2^ for Breakfasts, Lunches, and Dinners **eFigure 9.** Marginal *R*^2^ Over Time for Explaining Postprandial Glucose Variance Across Various Models of Increasing Complexity **eTable 2.** Model Fit of Different Multilevel Functional Regression Models Incorporating Different Predictor Variables **eFigure 10.** Estimated Pearson Residuals Along Time for Breakfasts, Lunches, and Dinners **eTable 3.** Marginal Coverage of the Estimated Confidence Bands Assessed Through Bootstrap Resampling **eTable 4.** List of Menus Classified According to Their GL Value
eReferences

Supplement 2.
Data Sharing Statement
