# Supplementary material for: Age, Sex, BMI, Meal Timing, and Glycemic Response to Meal Glycemic Load
Source: JAMA Netw Open. 2025 Sep 23;8(9):e2533193. doi: 10.1001/jamanetworkopen.2025.33193 (PMC12457981; doi:10.1001/jamanetworkopen.2025.33193)
Supplement: Supplement 1. — eMethods. eFigure 1. Study Procedures eFigure 2. Flowchart of the Recruitment Process and Study Phases eTable 1. Participants’ Daily Intake and GL Distribution eFigure 3. Effect of BMI and HbA1c on Postprandial Glucose Trajectories eFigure 4. Effect of Meal Timing on Postprandial Glucose Trajectories eFigure 5. Effect of GL on Postprandial Glucose Across Different Age Values eFigure 6. Effect of GL on Postprandial Glucose Across Different BMI Values eFigure 7. Percentage of Postprandial Time Spent Above 130 mg/dL Depending on GL, BMI, and Age for Each Meal eFigure 8. Estimated Joint R2 for Breakfasts, Lunches, and Dinners eFigure 9. Marginal R2 Over Time for Explaining Postprandial Glucose Variance Across Various Models of Increasing Complexity eTable 2. Model Fit of Different Multilevel Functional Regression Models Incorporating Different Predictor Variables eFigure 10. Estimated Pearson Residuals Along Time for Breakfasts, Lunches, and Dinners eTable 3. Marginal Coverage of the Estimated Confidence Bands Assessed Through Bootstrap Resampling eTable 4. List of Menus Classified According to Their GL Value eReferences [file jamanetwopen-e2533193-s001.pdf]

Supplementary Online Content

Calvo-Malvar M, Lado-Baleato O, Cao Ríos A, et al. Age, sex, BMI, meal timing, and glycemic response to meal glycemic load. *JAMA Netw Open*. 2025;8(9):e2533193. doi:10.1001/jamanetworkopen.2025.33193

eMethods

eFigure 1. Study Procedures

eFigure 2. Flowchart of the Recruitment Process and Study Phases

eTable 1. Participants' Daily Intake and GL Distribution

eFigure 3. Effect of BMI and HbA_{1c} on Postprandial Glucose Trajectories

eFigure 4. Effect of Meal Timing on Postprandial Glucose Trajectories

eFigure 5. Effect of GL on Postprandial Glucose Across Different Age Values

eFigure 6. Effect of GL on Postprandial Glucose Across Different BMI Values

eFigure 7. Percentage of Postprandial Time Spent Above 130 mg/dL Depending on GL, BMI, and Age for Each Meal

eFigure 8. Estimated Joint R^2 for Breakfasts, Lunches, and Dinners

eFigure 9. Marginal R^2 Over Time for Explaining Postprandial Glucose Variance Across Various Models of Increasing Complexity

eTable 2. Model Fit of Different Multilevel Functional Regression Models Incorporating Different Predictor Variables

eFigure 10. Estimated Pearson's Residuals Along Time for Breakfasts, Lunches, and Dinners

eTable 3. Marginal Coverage of the Estimated Confidence Bands Assessed Through Bootstrap Resampling

eTable 4. List of Menus Classified According to Their GL Value

eReferences

This supplementary material has been provided by the authors to give readers additional information about their work.

© 2025 Calvo-Malvar M et al. *JAMA Network Open*.

eMethods

Procedures

eFigure 1. Study procedures

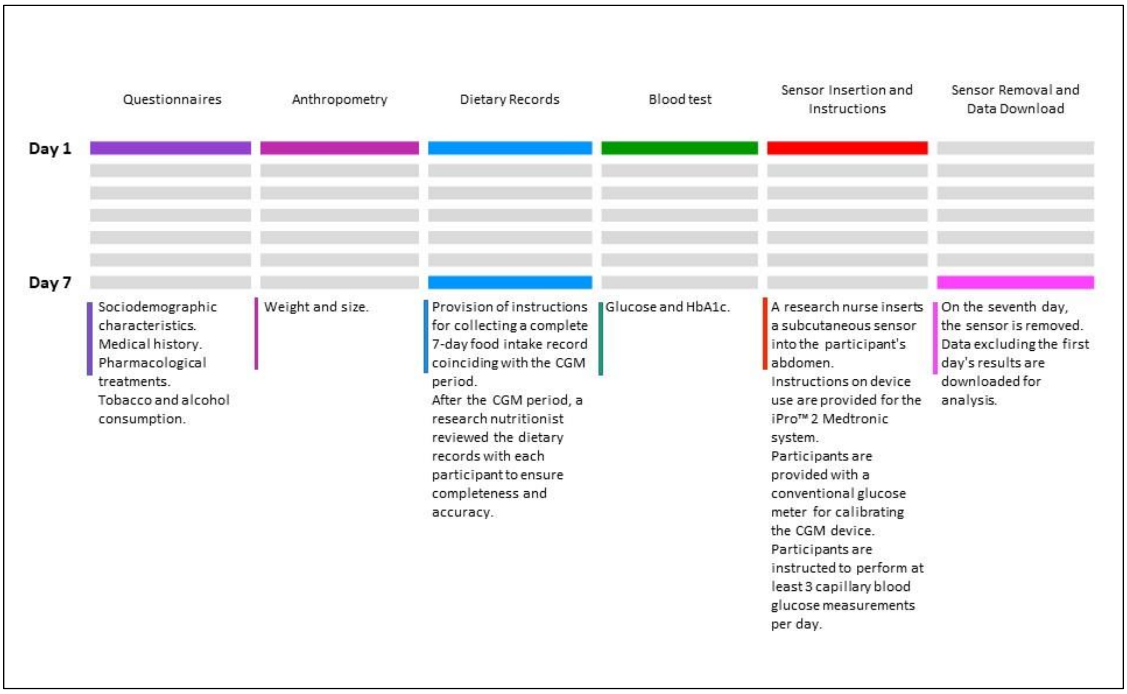

© 2025 Calvo-Malvar M et al. *JAMA Network Open*.

Glycemic response

The iPro™ system continuously measured interstitial glucose concentration every 5 minutes within a range of 40 to 400 mg/dL, storing the data in the CGM device. This system does not display real-time glucose values to prevent participant behaviour modification. Participants were also provided with a conventional glucose meter (One Touch Verio Pro; LifeScan, Milpitas, CA, USA), lancets, and compatible test strips for the purpose of calibrating the CGM device. To ensure data reliability and quality, participants were instructed to perform at least three capillary blood glucose measurements per day, typically before main meals. These capillary blood glucose readings were used to calibrate the CGM device. Monitoring data from days with fewer than three capillary blood glucose measurements were excluded from the analysis to maintain data integrity.

Dietary intake

To assess dietary intake, participants completed a 7-day food record that coincided with the CGM period. Detailed information was recorded regarding the types and amounts of foods and beverages consumed, including preparation methods, ingredients, sauces, and mealtimes. Participants were instructed to maintain their usual diet and daily routine during the study period.

After the CGM period, a research nutritionist reviewed the dietary records with each participant to ensure completeness and accuracy. For records that were incomplete or unreliable, a visual method using a food portion book was employed to quantify food amounts.[1]

The energy content, nutrient composition, fibre, glycemic index (GI), and glycemic load (GL) of the consumed foods and beverages were calculated using the DIAL nutritional

© 2025 Calvo-Malvar M et al. *JAMA Network Open*.

analysis software,[2] which utilizes the Food Composition Tables of the Department of Nutrition at the Complutense University of Madrid.[3] Energy and nutrient intake were expressed as daily averages.

Glycemic indices

The GI compares the impact of a food's carbohydrates on blood glucose levels to a standard glucose reference.[4] The GL takes into account both the quantity and quality of carbohydrates in a food by multiplying the food's GI by the amount of available carbohydrate consumed.

GI values were calculated by multiplying the available carbohydrate content of each food by its respective GI and summing the products, divided by the total carbohydrate intake. Glucose was used as the reference with a GI of 100.[4,5]

GL was calculated as (total available carbohydrate intake x dietary GI) / 100.[6]

Anthropometric measures

Anthropometric measures were conducted in triplicate during the initial visit. Participants were weighed in light clothing using a calibrated SECA® 701 model Class III digital scale (Hamburg, Germany) with a precision of 0.1 kg. Height was measured without shoes using a portable SECA® 213 stadiometer (Hamburg, Germany) with a precision of 0.1 cm. Waist circumference (narrowest point between the bottom of the ribcage and the top of the iliac crest) and hip circumference (point of greatest gluteal prominence) were measured using a Seca 201 circumference tape measure (Hamburg, Germany). Body mass index (BMI) was calculated as weight (kg) divided by height squared (m^2).

Assessment of physical activity

© 2025 Calvo-Malvar M et al. *JAMA Network Open*.

Physical activity was assessed using the International Physical Activity Questionnaire (short form).[7] The questionnaire estimated the amount of physical exercise in METs/hour/week. Participants who met the criteria of engaging in at least 30 minutes of moderate activity, five days a week, or at least 20 minutes of vigorous activity, three days a week, or achieving a minimum of 450 MET-min/week were classified as physically active.

Laboratory analysis

All laboratory analyses were performed on the same day that samples were obtained at the University Clinical Hospital of Santiago. The extractions took place between 8:00 and 10:00 a.m. after a 10-14 hour fast. Participants were instructed to remain seated for at least 5 minutes prior to the blood draw, with venous stasis limited to less than 2 minutes.

Serum glucose levels were measured using the oxidase-peroxidase method with a clinical chemistry system Advia 2400 (Siemens Healthcare Diagnostics). HbA1c was determined using high-performance liquid chromatography with a Menarini Diagnostics HA-8160 analyser, with results reported in DCCT units.[8] Aliquots of the samples were stored at -80°C for potential future studies.

Statistical analysis

The data were presented as either mean ± standard deviation or median with interquartile ranges depending on the distribution of the variables. Categorical variables were expressed as absolute frequency and percentage.

To assess changes over time in glucose concentrations, Multilevel Functional Regression Models were used. This statistical approach is specifically designed to analyse data that are observed over time and characterized by complex patterns of variability.[9] It allows

© 2025 Calvo-Malvar M et al. *JAMA Network Open*.

studying how curves of the variable of interest change over time and how these curves are influenced by different factors or covariates.

The model combines two statistical approaches: functional data analysis and mixed-effects models. Functional data analysis focuses on analyzing functions (curves) rather than discrete data points, facilitating the description and comparison of glucose concentration trajectories over time. On the other hand, mixed-effects models take into account not only the variability between individuals but also within individuals, which improves the precision of the estimates.

In summary, the Multilevel Functional Regression Model is a powerful tool for examining how glucose concentration curves evolve over time, while simultaneously accounting for inter- and intra-individual variability. This framework allows for the evaluation of the effects of key predictors (e.g., GL, BMI, age, and sex) on postprandial glucose trajectories.

Model Specification

Considering a 3-hour period after each meal (i.e., $t \in [0, 180]$ minutes), we evaluated the interstitial glucose concentration (IGC) over time for each individual $i \in \{1, ..., n = 514\}$ and each day $j \in \{1, ..., n_i\}$ (with $n_i \in \{2, 3, 4, 5\}$ being the number of monitoring days available for each individual), The general model for breakfast, lunch, and dinner was specified as:

$$IGC_{i,j}(t) = \beta_0(t) + GL_{i,j}\beta_1(t) + BMI_i\, \beta_2(t) + age_i\beta_3(t) + sex_i\beta_4(t) + \mu_i(t) + \varepsilon_{i,j}(t),$$

where $\beta_0(t)$ represents the common baseline curve for all individuals and days, $GL_{i,j}$ is the glycemic load of the meal for each individual i on each occasion j, and BMI_i, age_i, and sex_i are the BMI, age, and sex of each individual i, with $\beta_1(t)$, $\beta_2(t)$, $\beta_3(t)$, and $\beta_4(t)$ being the corresponding coefficients for these variables over time. $\mu_i(t)$ represents the error

© 2025 Calvo-Malvar M et al. *JAMA Network Open*.

associated with interindividual variability and $\varepsilon_{i,j}(t)$ represents the residual error associated with intraindividual variability and the statistical model used.

The errors $\mu_i(t)$ and $\varepsilon_{i,j}(t)$ were assumed to follow independent normal distributions with a mean of zero and a variance-covariance matrix of σ_μ and σ_ε for all time t, respectively.

$\mu_i(t) \sim N(0, \sigma_\mu)$

$\varepsilon_{i,j}(t) \sim N(0, \sigma_\varepsilon)$

From the proposed model, to assess the individual effect of each variable on glucose concentration while holding other variables constant, the coefficient β value is multiplied by the corresponding variable value and added to the β_0 coefficient that represents the glucose concentration at that time. This allowed us to appreciate, through the curves, the magnitude and timing of each studied variable's influence on glucose concentration according to the type of main meal.

This statistical model is fitted in two steps Fast Univariate Inference (FUI) procedure:

1. Point-wise mixed modelling: A separate GLMM is fitted at every 5-minute grid point $t \in [0, 180]$ min; no basis expansion is used at this stage.
2. Smoothing of coefficient curves. Each raw coefficient vector $\hat{\beta}_r(s)$ is smoothed with cubic B-splines:

$$\hat{\beta}_r(s) = \mathbf{b}(s)^T(\mathbf{B}^T\mathbf{B} + \lambda_r\mathbf{P})^{-1}\mathbf{B}^T\hat{\boldsymbol{\beta}}_r$$

where b(s) contains K = 15 equally spaced interior knots. The smoothing parameter λ_r is chosen by REML. Sensitivity checks with $K \in \{10, 20\}$ altered fitted curves by < 0.5 mg/dL, confirming robustness.

Functional R^2 Metrics

© 2025 Calvo-Malvar M et al. *JAMA Network Open*.

The explanatory capacity of the multilevel functional model was evaluated using functional R^2 metrics,[10] which quantify the proportion of variance in glucose concentrations explained by the model across the entire postprandial period. Two distinct measures of functional R^2 were used: joint R^2 and marginal R^2.

Joint R^2 incorporates both fixed effects (e.g., GL, BMI, age, and sex) and random effects (individual-specific variability). Joint R^2 is particularly useful for evaluating the overall fit of the model. This measure provides a more comprehensive assessment of how well the model explains the observed data, especially in contexts where individual-specific factors are important, such as in longitudinal studies or when dealing with heterogeneous populations.

Marginal R^2 focuses exclusively on fixed effects, isolating the explanatory power of predictor variables (e.g., GL, BMI, age, and sex) while excluding individual-specific variability. This measure highlights the direct influence of covariates on glucose concentrations, independent of random effects. Marginal R^2 is especially useful for evaluating the predictive strength of specific factors and identifying their relative importance in explaining postprandial glucose variance.

By leveraging these metrics, we assessed not only the overall performance of the model but also the specific contribution of predictor variables to glucose dynamics during the postprandial period.

Diagnostics beyond functional R^2

Three complementary diagnostics tests were performed: leave-one-day-out MAE, Pearson's residual, and 95% joint confidence bands for all coefficient curves.

Leave-one-day-out MAE, for each participant i we remove all observations from study day d, refit the model, and compute the integrate absolute error:

© 2025 Calvo-Malvar M et al. *JAMA Network Open*.

$$AE_{ij}^{-d} = \int_0^{180} |Y_{ij}(s) - \hat{Y}_{ij}^{-d}(s)| ds$$

Pearson residuals: point-wise Pearson residuals $r_{ij}(s) = (Y_{ij}(s) - \hat{\mu}_{ij}(s))/\hat{\sigma}(s)$ were plotted depending on time.

Bootstrap marginal-coverage test: to verify the analytic fast univariate inference variance formula attains the nominal marginal 95% parametric-bootstrap test:

1. Generate bootstrap data. For $b = 1, \dots, B$ with B = 1000.
   1.1. Draw new random-intercept curves $\tilde{u}_{0i}^{(b)}(s) \sim N\left(0, \hat{\sigma}_u^2(s)\right)$.
   1.2. Draw new residuals curves $\tilde{\varepsilon}_{ij}^{(b)}(s) \sim N\left(0, \hat{\sigma}_\varepsilon^2(s)\right)$.
   1.3. Form $\tilde{Y}_{ij}^{(b)}(s) = \hat{\beta}_0(s) + \boldsymbol{X}_{ij}^T \widehat{\boldsymbol{\beta}}(s) + \tilde{u}_{0i}^{(b)}(s) + \tilde{\varepsilon}_{ij}^{(b)}(s)$.
2. Refit the multilevel functional model to each $\tilde{Y}_{ij}^{(b)}(s)$ to obtain $\hat{\beta}_r^{(b)}(s), r = 1, \dots, p$.
3. Compute the coverage indicators. For every coefficient curve r and grid point s define

$$C_r^{(b)}(s) = 1\{\hat{\beta}_r^{(b)}(s) \in [\hat{\beta}_r(s) \pm z_{0.975} \sqrt{\widehat{Var}\{\hat{\beta}_r(s)\}}$$

4. Estimate the marginal coverage: $\widehat{Cov}_r(s) = \frac{1}{B} \sum_{b=1}^{B} C_r^{(b)}(s)$.

© 2025 Calvo-Malvar M et al. *JAMA Network Open*.

eFigure 2. Flowchart of the recruitment process and study phases

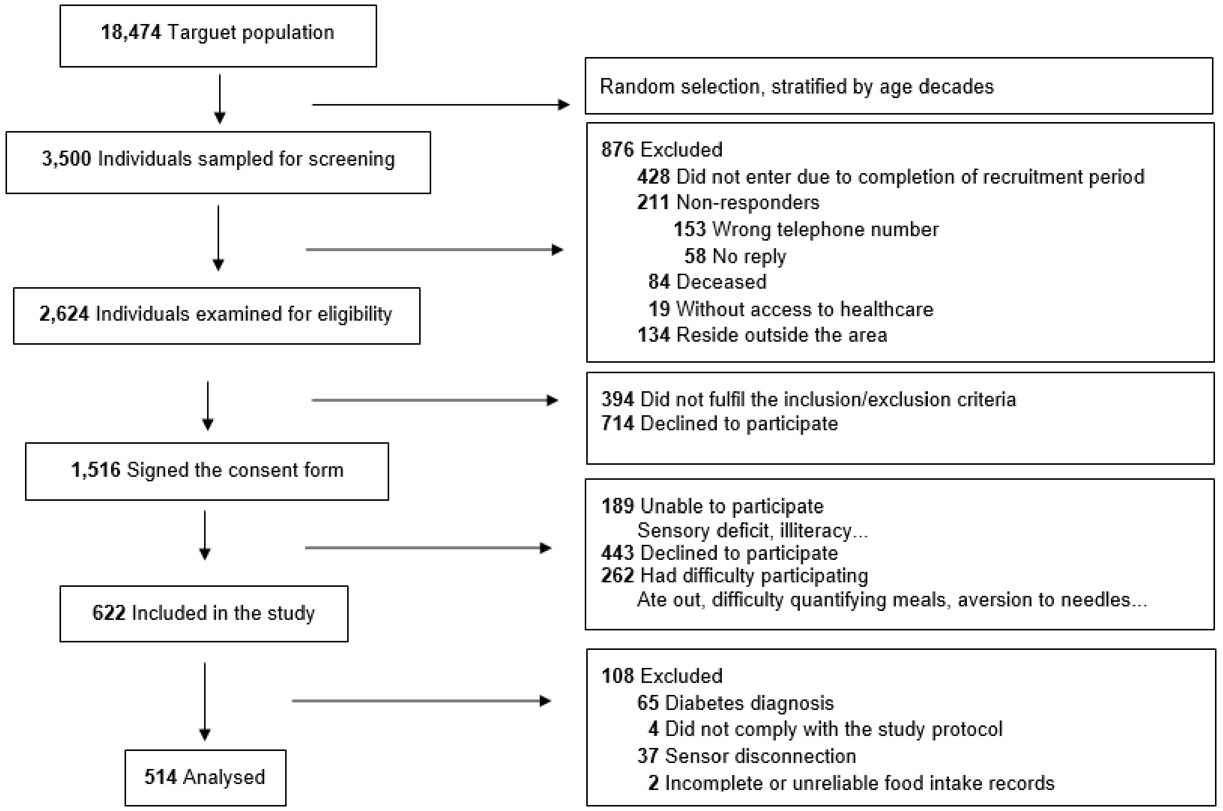

© 2025 Calvo-Malvar M et al. *JAMA Network Open*.

eTable 1. Participants' daily intake and GL distribution

	Minimum	1st quartile	Median	Mean	3rd quartile	Maximum
Kcal	448	1610	2054	2151	2564	6606
Women	448	1503	1887	1956	2313	6422
Men	484	1928	2388	2494	2949	6606
GL	5	76	104	113	142	420
Women	5	71	96	104	130	372
Men	8	86	121	128	160	420
GL	5	76	104	113	142	420
Breakfast	0	10	19	22	30	175
Lunch	0	22	36	41	55	236
Dinner	0	15	27	32	44	310

GI: glycemic index; GL: glycemic load

© 2025 Calvo-Malvar M et al. *JAMA Network Open*.

eFigure 3 illustrates the independent effects of BMI and glycated hemoglobin (HbA1c) on postprandial glucose response, as estimated by the multivariate functional mixed model, after adjusting for meal GL, age, sex, and meal timing.

© 2025 Calvo-Malvar M et al. *JAMA Network Open*.

eFigure 3. Effect of BMI and HbA1c on postprandial glucose trajectories

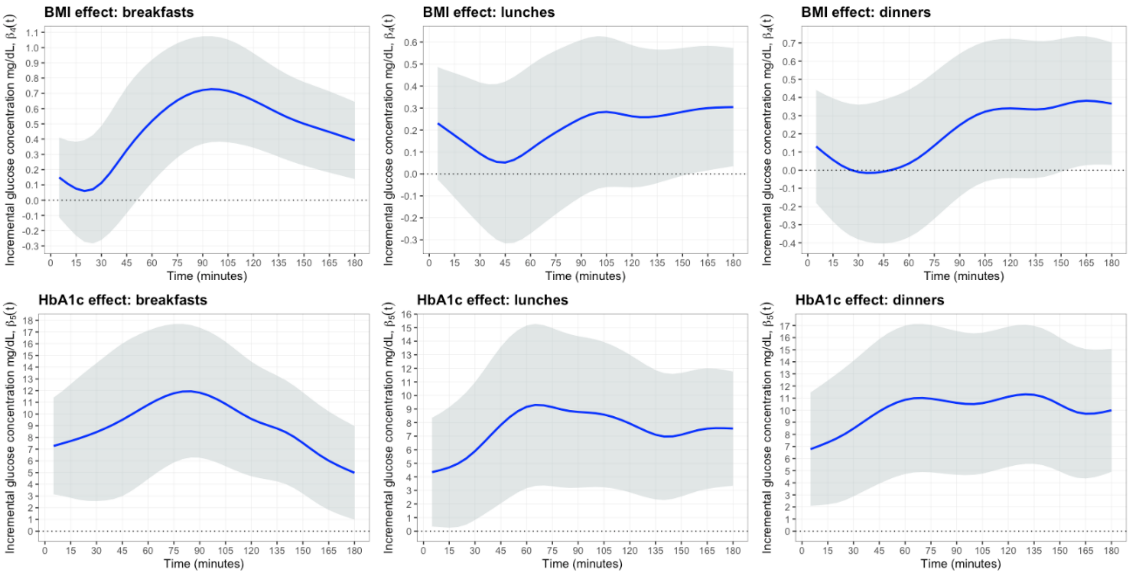

BMI = body mass index.

© 2025 Calvo-Malvar M et al. *JAMA Network Open*.

eFigure 4 depicts the independent effects of time elapsed since waking (time awake) and time remaining until going to bed (time to sleep) on postprandial glucose response. This analysis aims to ascertain the impact of circadian rhythm on postprandial glucose responses after adjusting for GL of meals, age, sex, BMI and HbA1c.

© 2025 Calvo-Malvar M et al. *JAMA Network Open*.

eFigure 4. **Effect of meal timing on postprandial glucose trajectories**

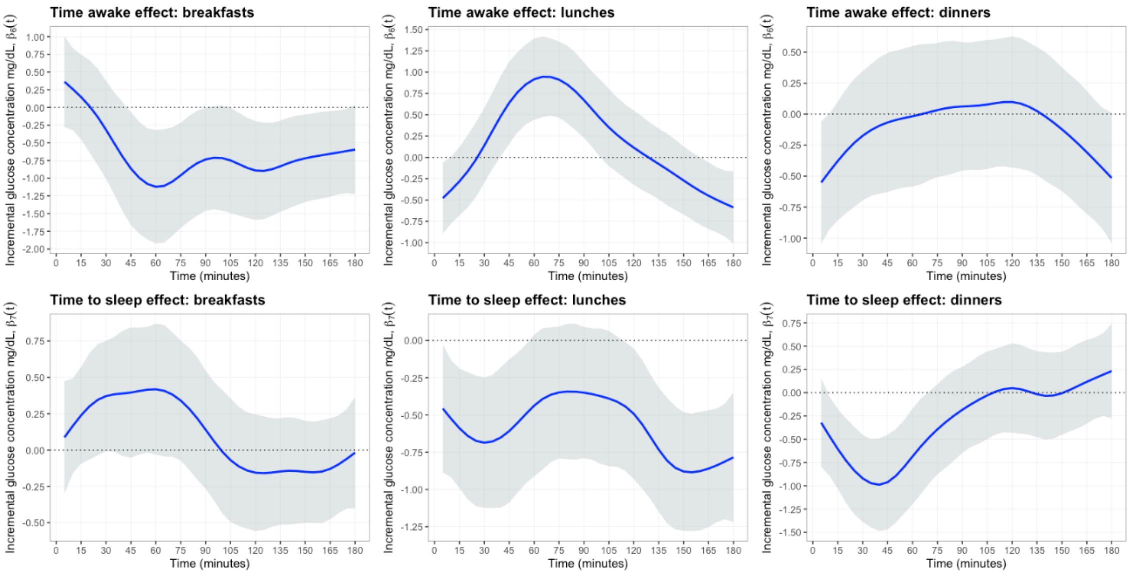

© 2025 Calvo-Malvar M et al. *JAMA Network Open*.

eFigure 5. **Effect of GL on postprandial glucose across different age values**

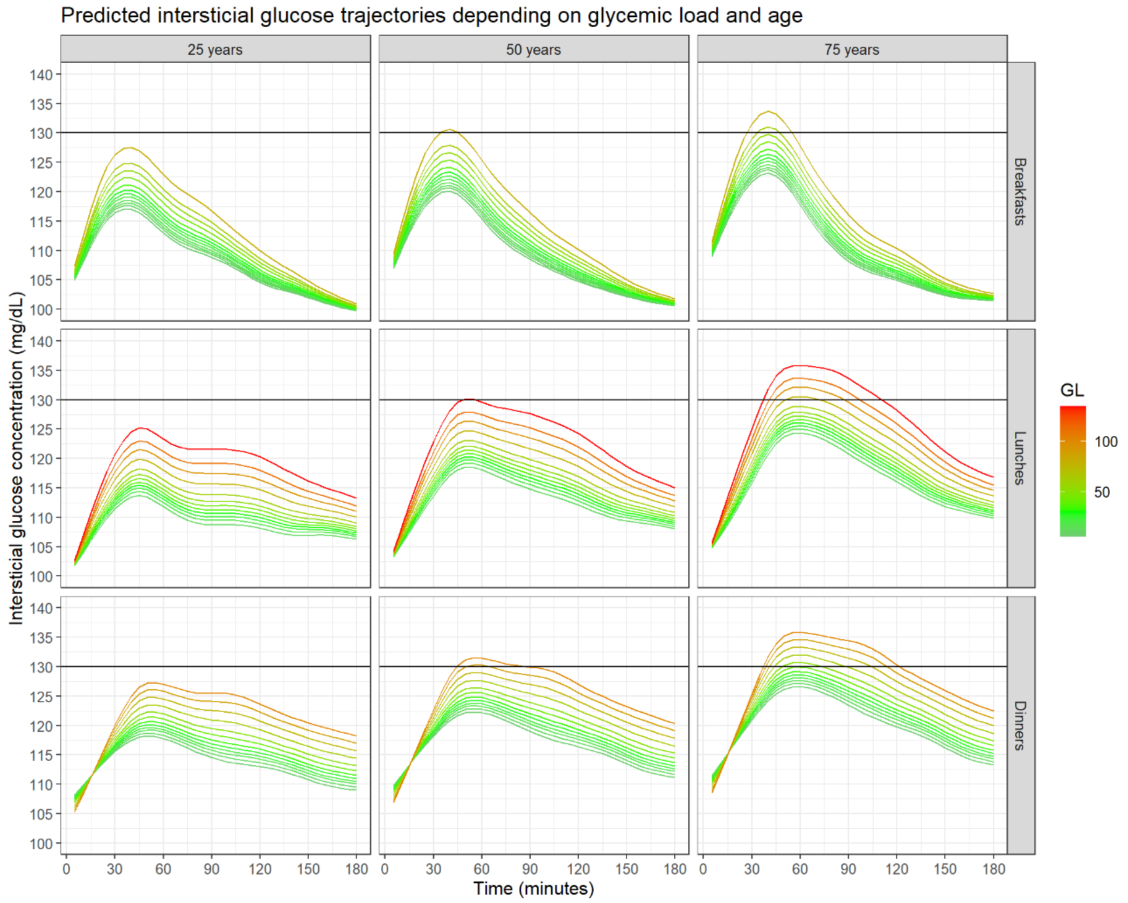

GL = glycemic load.

© 2025 Calvo-Malvar M et al. *JAMA Network Open*.

eFigure 6. Effect of GL on postprandial glucose across different BMI values

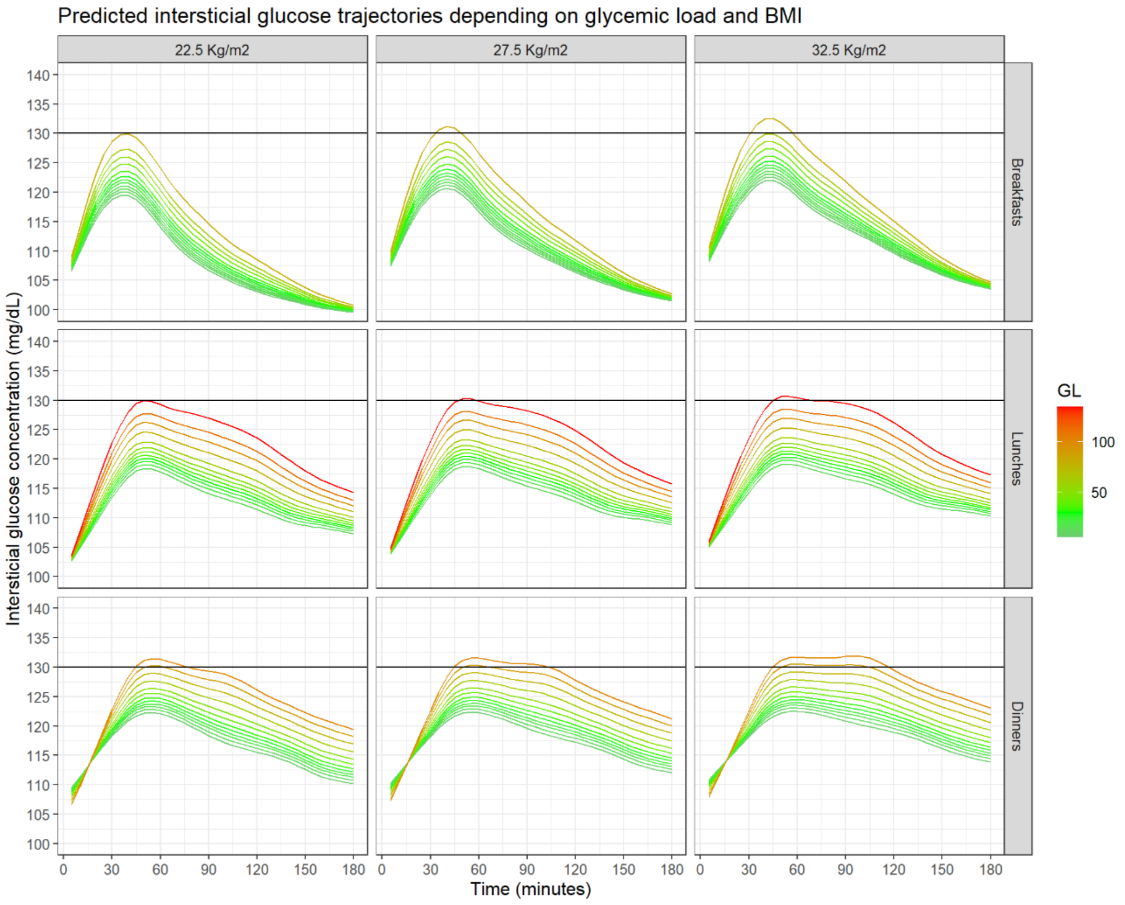

BMI = body mass index; GL = glycemic load.

© 2025 Calvo-Malvar M et al. *JAMA Network Open*.

eFigure 5 and eFigure 6 illustrate the predicted postprandial glucose responses to meals with varying GL, taking into account the effects of age and BMI. The colored continuous lines represent different levels of GL, based on specific percentiles (deciles, 95th, 97.5th, and 99th) calculated separately for breakfast, lunch, and dinner. Predictions were generated for a woman, with all other variables (HbA1c, time awake and time to sleep) held at their median values in the dataset to isolate the effects of GL, age, and BMI. For age, three representative values were considered: 25, 50, and 75 years. For BMI, the values selected were 22.5, 27.5, and 32.5 kg/m^2.

eFigure 7 shows the percentage of time a subject spends with postprandial blood glucose levels above 130 mg/dL, depending on age, BMI, and GL for breakfasts, lunches, and dinners. The predictions are made for a woman whose HbA1c is set to the median value of our sample (5.4%). Additionally, the times of waking up and going to sleep are fixed at the median values observed in the dataset for each meal. The GL values are set arbitrarily at 25, 50, and 75.

© 2025 Calvo-Malvar M et al. *JAMA Network Open*.

eFigure 7 **Percentage of postprandial time spent above 130 mg/dL depending on GL, BMI, and age for each meal**

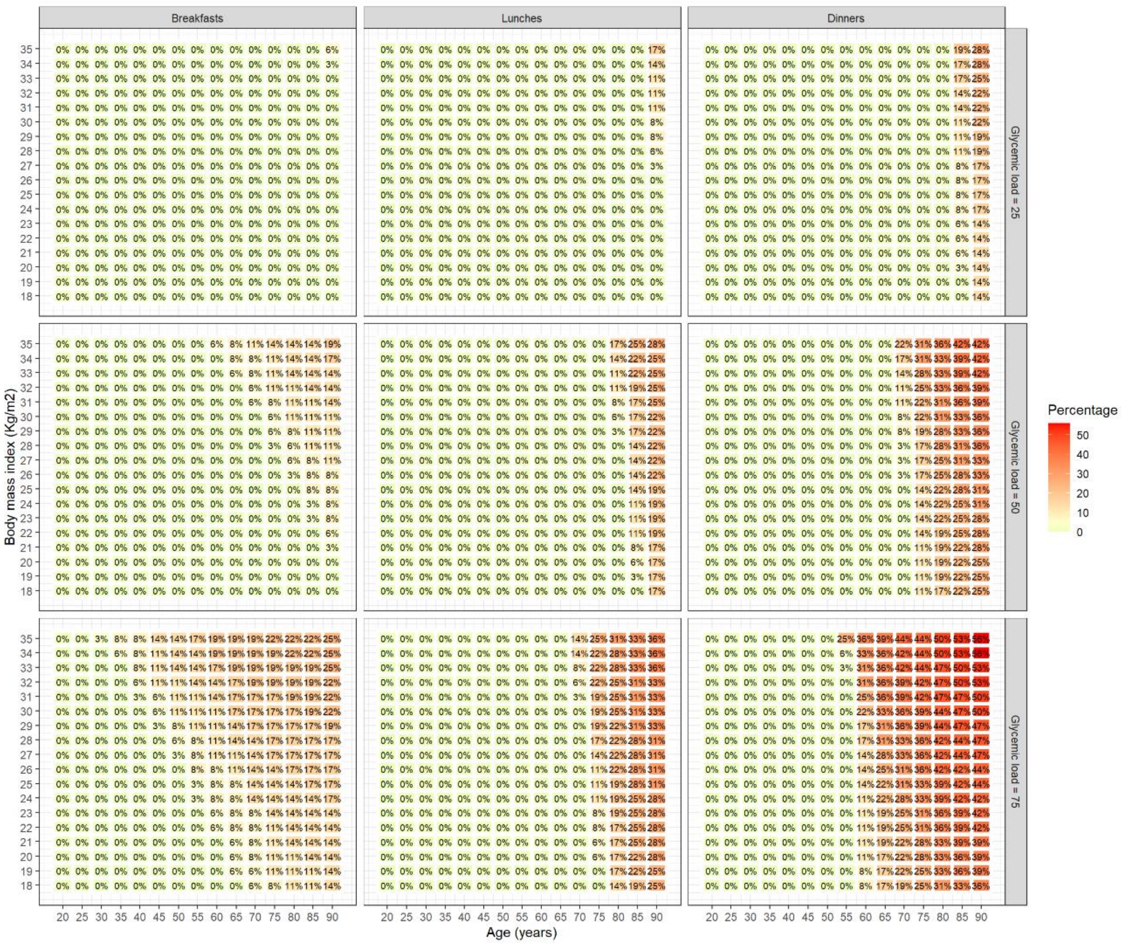

GL = glycemic load; BMI = body mass index.

© 2025 Calvo-Malvar M et al. *JAMA Network Open*.

The explanatory power of the multilevel functional model was evaluated using functional joint R^2 (R^2). eFigure 8 illustrates how R^2 values change over time following different meals: breakfasts, lunches, and dinners. For breakfasts, R^2 starts at a moderate level (0.44), peaks early ($R^2 = 0.50$ around 50 minutes), and then steadily declines, reflecting a rapid initial response with diminishing explanatory power over time. For lunches and dinners, R^2 begins at lower levels (0.28 and 0.31, respectively), peaks later at around 100 minutes, and then gradually decreases, reflecting a lower but more sustained response compared to breakfasts. These differences in R^2 values between meals indicate distinct postprandial glucose dynamics.

eFigure 8. Estimated joint R^2 for breakfasts, lunches and dinners

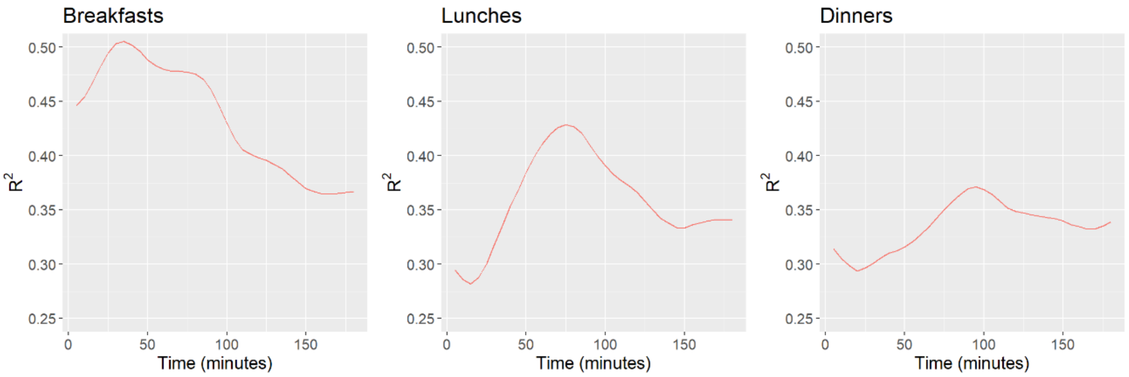

© 2025 Calvo-Malvar M et al. *JAMA Network Open*.

eFigure 9 presents the explanatory power (marginal R^2) of various models for postprandial glucose dynamics over time after breakfasts, lunches, and dinners. Each panel corresponds to a meal type, with R^2 values measuring how well the models' fixed effects explain glucose level variance at each time point. The models incorporate variables progressively: the baseline null model (orange), GL of meals (brown), GL and age (green), GL with age and and sex (blue), GL with age, sex, and BMI (turquoise), the model incorporating GL, age, sex, BMI, HbA1c (purple) and the full model (pink). Across all meals, marginal R^2 values increase shortly after eating, peak around 90 minutes, and then gradually decline. Incorporating variables such as GL, BMI, age, and sex consistently improves model performance, as shown by higher marginal R^2 values for the full model (pink line).

Breakfasts and lunches achieve the highest marginal R^2 values, indicating that postprandial glucose levels after these meals are better explained by the included variables. In contrast, dinners exhibit lower marginal R^2 values overall, suggesting that other unmeasured factors may have a greater influence on postprandial glucose dynamics during the evening.

© 2025 Calvo-Malvar M et al. *JAMA Network Open*.

eFigure 9. Marginal R^2 over time for explaining postprandial glucose variance across various models of increasing complexity

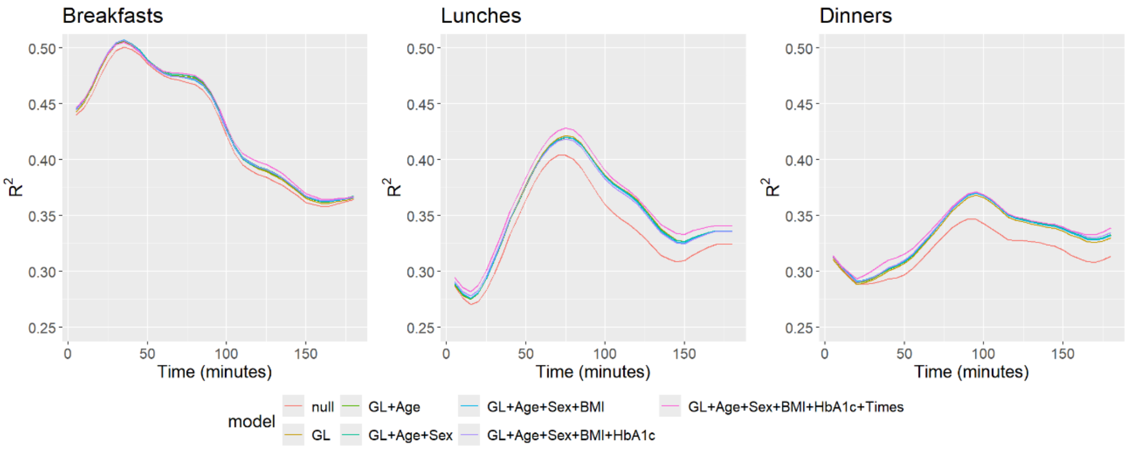

GL = glycemic load; BMI = body mass index.

© 2025 Calvo-Malvar M et al. *JAMA Network Open*.

The null model refers to the intercept-only model, while GL represents the glycemic load of meals, and BMI refers to body mass index.

eTable 3 summarizes the model fit measurements for multilevel functional regression models with increasing complexity by incorporating additional covariates for different meals. For the complete model, breakfasts exhibit the highest joint R^2, with marginal R^2 increasing from 0.000 in the null model to 0.079 when GL, BMI, age, and sex are included. Lunches show slightly lower joint R^2 values compared to breakfasts, with marginal R^2 rising from 0.000 in the null model to 0.093 in the complete model. Dinners display the lowest joint R^2, with marginal R^2 increasing from 0.000 in the null model to 0.067 in the complete model. The root mean square error (RMSE) of the functional model remains below 8.3 mg/dL, reflecting the model's accuracy in predicting glucose levels.

© 2025 Calvo-Malvar M et al. *JAMA Network Open*.

eTable 2. Model fit of different multilevel functional regression models incorporating different predictor variables.

Multilevel Functional Regression Model ($IGC_{i,j}(t) =$)	Joint R^2 (max.)	Marginal R^2 (max.)	RMSE
**Breakfasts**			
$\beta_0(t) + \mu_i(t) + \varepsilon_{i,j}(t)$		0.000	
$\beta_0(t) + GL_{i,j}\beta_1(t) + \mu_i(t) + \varepsilon_{i,j}(t)$		0.010	
$\beta_0(t) + GL_{i,j}\beta_1(t) + BMI_i\,\beta_2(t) + \mu_i(t) + \varepsilon_{i,j}(t)$		0.072	
$\beta_0(t) + GL_{i,j}\beta_1(t) + BMI_i\,\beta_2(t) + age_i\beta_3(t) + \mu_i(t) + \varepsilon_{i,j}(t)$		0.076	
$\beta_0(t) + GL_{i,j}\beta_1(t) + BMI_i\,\beta_2(t) + age_i\beta_3(t) + sex_i\beta_4(t) + \mu_i(t) + \varepsilon_{i,j}(t)$		0.078	
$\beta_0(t) + GL_{i,j}\beta_1(t) + BMI_i\,\beta_2(t) + age_i\beta_3(t) + sex_i\beta_4(t) + HbA1c_i\beta_5(t) + Time\_awake_i\beta_6(t) + Time\_to\_sleep_i\beta_7(t) + \mu_i(t) + \varepsilon_{i,j}(t)$	0.505	0.109	6.52
**Lunches**			
$\beta_0(t) + \mu_i(t) + \varepsilon_{i,j}(t)$		0.000	
$\beta_0(t) + GL_{i,j}\beta_1(t) + \mu_i(t) + \varepsilon_{i,j}(t)$		0.020	
$\beta_0(t) + GL_{i,j}\beta_1(t) + BMI_i\,\beta_2(t) + \mu_i(t) + \varepsilon_{i,j}(t)$		0.043	
$\beta_0(t) + GL_{i,j}\beta_1(t) + BMI_i\,\beta_2(t) + age_i\beta_3(t) + \mu_i(t) + \varepsilon_{i,j}(t)$		0.092	
$\beta_0(t) + GL_{i,j}\beta_1(t) + BMI_i\,\beta_2(t) + age_i\beta_3(t) + sex_i\beta_4(t) + \mu_i(t) + \varepsilon_{i,j}(t)$		0.093	
$\beta_0(t) + GL_{i,j}\beta_1(t) + BMI_i\,\beta_2(t) + age_i\beta_3(t) + sex_i\beta_4(t) + HbA1c_i\beta_5(t) + Time\_awake_i\beta_6(t) + Time\_to\_sleep_i\beta_7(t) + \mu_i(t) + \varepsilon_{i,j}(t)$	0.428	0.111	7.83
**Dinners**			
$\beta_0(t) + \mu_i(t) + \varepsilon_{i,j}(t)$		0.000	
$\beta_0(t) + GL_{i,j}\beta_1(t) + \mu_i(t) + \varepsilon_{i,j}(t)$		0.014	
$\beta_0(t) + GL_{i,j}\beta_1(t) + BMI_i\,\beta_2(t) + \mu_i(t) + \varepsilon_{i,j}(t)$		0.044	
$\beta_0(t) + GL_{i,j}\beta_1(t) + BMI_i\,\beta_2(t) + age_i\beta_3(t) + \mu_i(t) + \varepsilon_{i,j}(t)$		0.061	
$\beta_0(t) + GL_{i,j}\beta_1(t) + BMI_i\,\beta_2(t) + age_i\beta_3(t) + sex_i\beta_4(t) + \mu_i(t) + \varepsilon_{i,j}(t)$		0.067	
$\beta_0(t) + GL_{i,j}\beta_1(t) + BMI_i\,\beta_2(t) + age_i\beta_3(t) + sex_i\beta_4(t) + HbA1c_i\beta_5(t) + Time\_awake_i\beta_6(t) + Time\_to\_sleep_i\beta_7(t) + \mu_i(t) + \varepsilon_{i,j}(t)$	0.372	0.087	8.432

R^2 = r-squared; RMSE = root mean square error; IGC = interstitial glucose concentration over time for each individual and each day; max. = maximum; GL = glycemic load; BMI = body mass index.

© 2025 Calvo-Malvar M et al. *JAMA Network Open*.

Median MAE for breakfast was 5.5 mg/dL (IQR 3.4, 9.1), for lunches 6.25 mg/dL (IQR 3.7, 9.8), and for dinners 6.12 mg/dL (IQR 3.86, 9.35). This values where within the ISO 15197 accuracy target of ±10 mg/dL.

We found no unmodeled heteroscedasticity on the Pearson residuals neither cluster of points (unmodelled within day dependence) (eFigure 10). No dependence was found for any time point on model's residuals.

As can be seen in eTable 4 marginal coverage of the confidence bands for each variable is close to the nominal level of 95%.

© 2025 Calvo-Malvar M et al. *JAMA Network Open*.

eFigure 10. Estimated Pearson's residuals along time for breakfasts, lunches and dinners

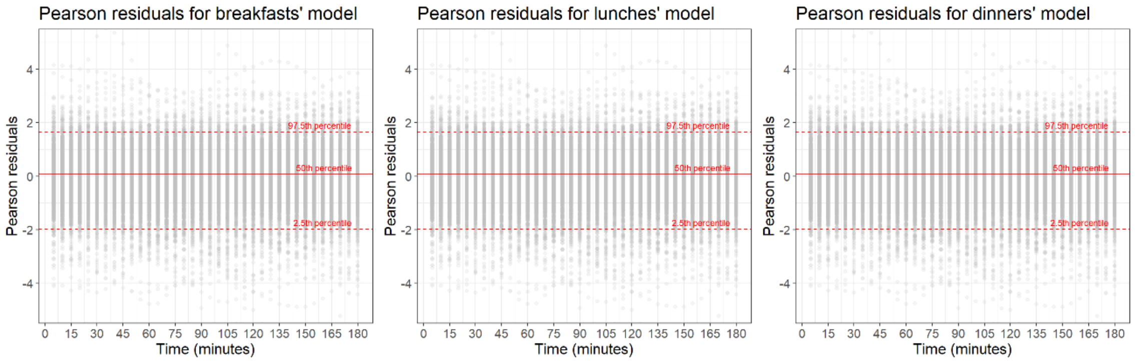

© 2025 Calvo-Malvar M et al. *JAMA Network Open*.

eTable 3. Marginal coverage of the estimated confidence bands assessed through bootstrap resampling

	Breakfasts	Lunches	Dinners
Intercept	97.5 (1.8)	98.8 (4.5)	99.7 (2.9)
Glycemic load	93.2 (11.8)	92.1 (5.6)	95.3 (10.9)
Age	96.8 (1.6)	97.9 (0.50)	96.8 (1.5)
Gender	98.8 (1.7)	98.9 (1.7)	97.9 (0.85)
BMI	94.7 (2.3)	96.8 (1.9)	96.9 (0.6)
HbA1c	93.9 (1.1)	91.8 (1.8)	95.9 (0.4)
Time awake	91.5 (1.4)	94.4 (1.6)	91.5 (16.9)
Time to sleep	92.4 (1.7)	94.5 (1.8)	92.2 (11.4)

© 2025 Calvo-Malvar M et al. *JAMA Network Open*.

eTable 4. List of menus classified according to their GL value

GL	Foods
	**Beakfasts**
**10**	200g skimmed milk, 5g instant coffee, 30g whole wheat bread, and 10g orange marmalade.
**20**	200g orange juice, 125g yogurt, and 32g whole wheat biscuits.
**30**	35g breakfast cereal, 325g semi-skimmed milk, and 15g chocolate powder.
**40**	200g skimmed milk, 15g chocolate powder, and 90g croissant.
**50**	250g yogurt, 45g breakfast cereal, and 25g sugar.
**60**	20g unsweetened cocoa powder, 35g sugar, 400g whole cow's milk, and 100g bread.
**70**	250g skimmed milk, 10g sweetened cocoa powder, 4g sugar, and 140g biscuits.
**80**	354g semi-skimmed coffee with no sugar, 110g bread, and 90g biscuits.
**90**	180g corn cakes, 160g tuna, and 5g cream cheese.
**100**	200g whole cow's milk, 10g sweetened cocoa powder, and 150g wheat and chocolate-based breakfast cereal.
**150**	300g semi-skimmed milk, 220g corn-based breakfast cereal, 5g chocolate powder, and 5g sugar.
**200**	350g milk, 200g honey-flavored cereal, 5g chocolate powder, 160g sponge cake, and 200g orange juice.
	**Lunches**
**10**	235 g stewed lentils.
**20**	250 g boiled green beans, 200 g grilled chicken fillet, 125 g yogurt, 50 g shelled walnuts, 50 g chocolate, 50 g liquid coffee, and 12 g sugar.
**30**	100 g tomato, 120 g lettuce, 10 g oil, 5 g salt, 5 g vinegar, 95 g french fries, 55 g veal chop, 65 g bread, 75 g vermouth, and 300 g red wine.
**40**	273 g boiled potatoes, 95 g pork liver, 10 g sunflower oil, 47 g bread, and 50 g ice cream cake.
**50**	233 g lentils with chorizo sausage, 159 g meat stew, 68 g bread, 78 g strawberries, and 200 g orange juice.
**60**	34340 g potatoes with cod, 113 g bread, and 96 g mandarin.
**70**	358 g rice with beef, 400 g boiled green beans, and 70 g bread.
**80**	300 g seafood paella, 105 g bread, 63 g turkey breast deli meat, and 125 g plain yogurt.
**90**	400 g roasted chicken, 130 g palmiers, and 400 g lemon soda.
**100**	1175 g noodles with chicken, 150 g red wine, and 180 g banana.
**150**	300 g corn tortilla, 200 g semi-fat pork meat, 183 g lettuce and tomato salad, and 100 g liquid decaffeinated coffee.
**200**	177 g tuna empanada, 60 g bread, 20 g cream cheese, 30 g anchovy, 60 g chorizo sausage, 590 g seafood paella, 182 g decaffeinated coffee with milk and sugar, 5 g sugar, and 800 g orange Fanta.
	**Dinners**
**10**	200 g semi-skimmed milk, 20 g wholemeal cookies, and 18 g toasted wholemeal bread.
**20**	251 g Spanish tortilla with potatoes, 89 g lettuce salad, 50 g Arzúa cheese, and 185 g coffee with milk and sugar.
**30**	130 g tomato salad, 75 g cucumber, 40 g semi-cured cheese, 60 g sliced cooked ham, and 75 g bread.
**40**	100 g European hake, 10 g olive oil, 79 g bread, 120 g cherries, and 223 g apple.
**50**	142 g ham and cheese pizza and 330 g lemon-lime flavored soda.
**60**	311 g chocolate cake and 20 g cookies.
**70**	300 g noodle soup, 138 g baked hake, 251 g tomato salad, 40 g bread, and 213 g Galician almond cake.
**80**	478 g rice pudding, 343 g figs, and 79 g shelled walnuts.
**90**	360 g milk chocolate, 27 g bread, and 22 g honey cereal.
**100**	80 g chorizo sausage, 100 g bread, 50 g small ring-shaped cookies, 137 g Carnival pastries, 100 g liquid coffee, and 20 g sugar.
**150**	75 g liquid coffee, 100 g skimmed milk, 1 g stevia sweetener, 200 g digestive biscuits, and 175 g cookies.
**200**	50 g white baguette bread, 350 g spaghetti, 175 g lemon soda, and 62 g donuts.

GL = glycemic load

© 2025 Calvo-Malvar M et al. *JAMA Network Open*.

eReferences

1. Russolillo Femenías G, Marques-Lopes I. Guía visual de alimentos: Álbum fotográfico de porciones de alimentos; 2011.
2. Ortega RM, López-Sobaler AM, Carvajales PA, Requejo AM, Aparicio A, Molinero LM. Programa Dial v. 3.3.5.0. 2016. Available online: https://www.alceingenieria.net/nutricion/descarga.htm (accessed on 5 April 2021).
3. Ortega RM, López-Sobaler AM, Requejo AM, Andrés P (2008) La composición de los alimentos. Herramienta básica para la valoración nutricional. Editorial Complutense, Madrid
4. Wolever TM, Jenkins DJ, Jenkins AL, Josse RG. The glycemic index: methodology and clinical implications. *Am J Clin Nutr* 1991;**54(5)**: 846–54.
5. Chew I, Brand JC, Thorburn AW, Truswell AS. Application of glycemic index to mixed meals. *Am J Clin Nutr* 1988;**47**:53–6.
6. Barclay AW, Brand-Miller JC, Wolever TM. Glycemic index, glycemic load, and glycemic response are not the same. *Diabetes Care* 2005;**28**: 1839–1840.
7. The-IPAQ-Group International Physical Activity Questionnaire. Available at: https://sites.google.com/site/theipaq/home. Accessed February 2023.
8. Hoelzel W, Weykamp C, Jeppsson JO, et al. IFCC reference system for measurement of haemoglobin HbA 1C in human blood and the National Standardization Schemes in the United States, Japan, and Sweden: a method-comparison study. *Clin Chem* 2004;**50**:166–74.
9. Matabuena M, Karas M, Riazati S, Caplan N, Hayes PR. Estimating Knee Movement Patterns of Recreational Runners Across Training Sessions Using Multilevel Functional Regression Models. *Am Stat* 2023;**77(2)**:169–81.
10. Matabuena M, Sartini J, Gude, F. Multilevel functional data analysis modeling of human glucose response to meal intake. *arXiv* preprint arXiv 2024; **2405**:14690.

© 2025 Calvo-Malvar M et al. *JAMA Network Open*.
