# Supplementary material for: Age, Sex, BMI, Meal Timing, and Glycemic Response to Meal Glycemic Load
Source: JAMA Netw Open. 2025 Sep 23;8(9):e2533193. doi: 10.1001/jamanetworkopen.2025.33193 (PMC12457981; doi:10.1001/jamanetworkopen.2025.33193)
Supplement: Supplement 2. — Data Sharing Statement [file jamanetwopen-e2533193-s002.pdf]

Data Sharing Statement

Calvo-Malvar. Age, Sex, BMI, Meal Timing, and Glycemic Response to Meal Glycemic Load. *JAMA Netw Open*. Published September 23, 2025. doi:10.1001/jamanetworkopen.2025.33193

Data
Data available: Yes
Data types: Deidentified participant data
How to access data: The datasets generated and analyzed during the current study will be made available through a publicly accessible repository on publication at the Runa Digital Repository (runa.sergas.gal). To gain access, data requestors will need to sign a data access agreement. Proposals should be directed to francisco.gude@usc.es.
When available: With publication

Supporting Documents
Document types: None

Additional Information
Who can access the data: Researchers whose proposed use of the data has been approved
Types of analyses: Meta-analysis, systematic reviews, reviews.
Mechanisms of data availability: After approval of a proposal
